# Child Life under Queen Victoria

**Published:** 1897-03-13

**Authors:** 


### Child Life under Queen Victoria.

### V.—THE CLIMBING BOYS.

It was the children in the cotton factories who fought the battle of all children. Once it had become a principle of legislation that children should not be permitted to do work that interfered with their health, their growth, or their education, it was inevitable that the principle should be applied to all children. But with some classes of child workers it was less easy to replace infant with adult labourers than it was in the factories, In the collieries, as we saw, the plea was that full-grown men could not draw the coal hutches along the narrow passages so well as children; and, with regard to the chimney-sweeps, it was similarly urged that a man could not go up the average vent; the "climbing boy" was required. It took a hundred years of agitation—not to convince the nation that the child chimney-sweepers were cruelly treated, but that their sufferings could be dispensed with, and yet no one be the worse for it.

The climbing boy was an ancient institution. For centuries the only method of cleaning a chimney was to send a child up it to dislodge the soot by his passage and any efforts he might have to make to force his way through. Both boys and girls were employed at the unwholesome trade; some were sold to the master-sweeps by degraded parents, some were apprenticed by those harsh step-parents, the poor law guardians, and when supplies from these sources were insufficient, children were stolen from their homes to be set to this trade. Everything conspired to make these children outcasts. The very nature of their trade kept cleanly people from associating with them, and the hardships of their lives and the cruelties of their masters tended to brutalise them.

It was in 1760 that a letter addressed to the editor of the *Public Advertiser* first drew attention to the sufferings of these children. This aroused a good deal of sympathy. Jonas Hanway, who name is to be remembered as helping to found Sunday-schools, took up the question, formed an association which appealed to the master-sweeps to show such consideration as they could to their apprentices, and later, in 1785, published what he called a "Sentimental History of Chimney Sweepers," which exposed the cruelties from which the boys suffered, and declared the need of proper regulations. This had a certain result; that is, Parliament passed an Act forbidding master chimney-sweeps to take more than six apprentices, and insisting that these should not be under eight years of age! And this was all that was done for half a century.

Yet Jonas Hanway's labour had not been wasted. The Prince of Wales (George IV.—let this good work be remembered to his honour if in much else we cannot honour him!) took the lead in a "Society for Superseding the Necessity for Climbing Boys," which recommended the use of a machine for cleaning chimneys, and offered to present this to such master sweepers as could not afford to buy one. Had public opinion been in a healthy state nothing more in the way of legislation might have been needed. As it was, the well-meant action had little effect. At various times efforts were made to induce Parliament to take up the cause of the little chimney-sweeps, but without much result. In 1817 a Committee was appointed to inquire into their condition, which revealed enough to have aroused any nation to action. And yet nothing was done. In 1834 only was a Bill passed which forbade the employment of children under ten years of age. It also went so far as to make it criminal to send a child up a chimney while it was on fire, of which wickedness some masters had hitherto been guilty. It was also ordained that flues were not to measure less than fourteen inches by nine. How children got up flues smaller than this it would be difficult to say. Even these concessions were opposed. Lord Kenyon declared that the safety of the metropolis was endangered by it, and the fire insurance companies petitioned against it.

In 1840, however, a great improvement took place in the law. The lowest limit of age for apprentices was raised to sixteen, and even at that age they were not to be sent up the flues. A fine was to be inflicted on all who should compel or knowingly allow anyone under the age of twenty-one years to ascend or descend a chimney, or enter a flue, for the purpose of sweeping or cleaning it. By this time also the insurance companies had changed their opinions, and recommended their clients to have their chimneys swept by the machines instead of by children. Regulations were made as to the size and construction of chimneys in new buildings; but the real difficulty lay in the build of old chimneys, whose angles were too sharp to permit of any machine being effective. Yet it was not a case for compromise; the cruelties and degradation were too conspicuous. The children were forced with blows to go up the chimneys; if they stuck or seeemed to hesitate their masters pricked the soles of their feet, or scorched them with lighted straw. It was no infrequent thing for a climbing boy to stick in a chimney, and, unable to go up or down, be smothered to death. The children were sent up naked, and naked and unwashed they often passed the night among the soot-bags. Their filthy condition often resulted in an irritation of the skin, and "chimney-sweepers' cancer" was a recognised disease. Their moral condition was not less degrading than their physical one, and not a few lapsed into a life of crime.

After this time the habitual employment of children became less frequent in London, though the country at large was slow to take up the reformation, and even in the metropolis there were reactions. In 1853 we find Lord Shaftesbury promoting a Bill to amend the condition of chimney-sweepers, but unable to effect any result. It was not till 1864 that the "Chimney Sweepers' Regulation Act" was passed, which sentenced to imprisonment with hard labour any master who sent a boy up a chimney. Occasional evasions of this law occurred during the next ten years—the last death of a climbing boy in a flue occurred at Cambridge in February, 1875. The boy's master was sentenced to six months' hard labour for manslaughter; but public opinion was roused, and a final Act punished the employment of child-sweepers so severely that since that time the cruel and unnecessary practice has vanished from the land.

